# Effectiveness of a Nursing Educational Intervention in Adults to Promote Control Behaviors Against Dengue: Protocol for a Randomized Controlled Trial

**DOI:** 10.2196/54286

**Published:** 2024-02-23

**Authors:** Yolima Judith Llorente Pérez, Alba Luz Rodríguez-Acelas, Rita Mattiello, Wilson Cañon-Montañez

**Affiliations:** 1 Faculty of Nursing Universidad de Antioquia Medellín Colombia; 2 Nursing Program Universidad de Córdoba Montería Colombia; 3 Postgraduate Program in Epidemiology, Universidade Federal do Rio Grande do Sul Porto Alegre Brazil

**Keywords:** dengue, health education, health promotion, nursing care, prevention & control

## Abstract

**Background:**

The increase in dengue cases can be attributed to social, demographic, environmental changes, or community-driven factors. In this regard, different strategies have been established in health education, using educational interventions as necessary tools for the reduction of the disease with the aim of reinforcing and stimulating the prevention and control of dengue.

**Objective:**

This study aims to evaluate the effectiveness of a nursing educational intervention for dengue control.

**Methods:**

A randomized controlled trial will be conducted with adults living in rural areas and participating in health promotion and disease prevention programs. We will enroll 116 adults. Adults will be randomized 1:1, with 58 adults assigned to the educational intervention group and 58 to the usual care group. Participants will receive 4 sessions over the course of a month, 1 week apart, and will be followed up for 1 month after the end of the educational intervention. Nursing Outcome Classification labels will be used to measure the outcomes: risk control (1902) and participation in health care decisions (1606).

**Results:**

The participants in the intervention group are expected to achieve better dengue control behaviors than those in the usual care group.

**Conclusions:**

Risk factors are fostered by the community, largely caused by artificial reservoirs or unprotected tanks in homes; also, the lack of information hinders the identification of symptomatology and the poor implementation of effective measures, and the development of standardized educational strategies can contribute to efficient and cost-effective control of the disease.

**Trial Registration:**

ClinicalTrials.gov NCT05321264; https://clinicaltrials.gov/study/NCT05321264

**International Registered Report Identifier (IRRID):**

PRR1-10.2196/54286

## Introduction

The World Health Organization, in a report for 2023, reported that between 100 and 400 million cases of dengue occur annually [1]. It also indicates that in the Americas for the year 2021, there was an incidence of 123 cases per 100,000 inhabitants, with a case fatality rate of 0.034% [2].

Dengue in general has a global annual cost of US $8.9 billion, with an average annual cost per case of US $151 in 2013 [3] and a global burden of 1.14 million disability-adjusted life years [4]. The main burden on affected countries is the enormous number of hospitalizations and sick days, with older people being the most affected population [3,5]. For the economy, the negative effects are due to the high costs of controlling epidemics and absenteeism from work and school [6,7].

In addition, dengue generates indirect costs that are usually enormous. These costs are given by the consultations or medicines taken by the members of the family nucleus [8], or by the loss of income of the patients and their families due to hospitalizations and disabilities, which at the same time reflect a reduction in the supply of work from home [9,10], not to mention the distress that the patient and his family are exposed to, generated by the doubt, uncertainty, or disability that the disease can bring. Living in low socioeconomic strata in rural areas, urbanization, global warming, and increased human mobility may be factors contributing to the increased burden of the disease [11,12].

Studies in endemic countries, specifically in Latin America, reveal a low level of knowledge about dengue among the population [13]. In Peru, Gutiérrez and Montenegro-Idrogo [14] indicated that the population has a poor level of knowledge about dengue control and prevention. In Colombia, de Maria Cáceres-Manrique et al [15] reported that the knowledge of the participants about dengue is scarce, with favorable attitudes toward control but insufficient practices, and they also consider the disease to be something normal. Likewise, other studies reflect scarce knowledge about dengue, the adoption of risky practices that favor the development of the disease, and a lack of knowledge about ways to prevent dengue [16,17].

The increase of cases in these regions, apart from having behavioral and cognitive root components, such as the behavior and cultural patterns manifested by the population, essentially related to beliefs, customs, and traditions, which sometimes determine the circumstances in which the dengue virus vector lives, also presents factors that may favor the dynamics of the disease, such as socioeconomic (level of schooling, strata, overcrowding, and lack of public services), climatic, and political factors, where there is little participation of health institutions [18,19].

Research conducted in the study setting (Montería, Colombia) has related a lack of knowledge or inadequate knowledge, unfavorable attitudes, and inadequate behaviors in relation to dengue; in addition to this, people live in homes with characteristics that favor the development and multiplication of the vector, leading to recommendations for other interventions aimed at reducing contact with the vector, taking into account the conditions in which families live [20].

The presence or multiplication of dengue cases may be mostly preceded by the inadequate application of health behaviors in the management and control of the vector, having as a background the scarcity of knowledge for the appropriation and incorporation of sanitary measures in the different environments.

In view of the lack of knowledge and control measures for dengue, the literature points to several determining factors, such as sex [13], level of schooling, and socioeconomic level, since diseases such as dengue have a greater impact on low-income populations, where the conditions for the presence of vector breeding sites are more likely to occur. A low level of education generally coincides with a lack of knowledge of the disease. Other factors include residence in rural areas, occupation, overcrowding, and a lack of public services [19].

Other factors are lack of motivation and willingness, negative attitudes (low sense of belonging), inadequate habits or inappropriate cultural practices, lack of knowledge, difficult access to health services, lack of time, perception of risk, low involvement of health institutions, and limited economic and human resources allocated to public health infrastructures [21].

Thus, the evidence points to the increase in cases of dengue fever and cognitive and behavioral factors, which will be addressed in light of Nola Pender’s Health Promotion Model (HPM). This model proposes that people interact with the environment, trying to achieve an adequate state of health, schematizing the nexus between personal characteristics and experiences, knowledge, beliefs, and situational aspects linked to the health behaviors or behaviors that are intended to be achieved [22], it raises dimensions and relationships that participate to generate or modify the health-promoting behavior [22], being the positive response that is expected to the actions carried out.

**Figure 1:**
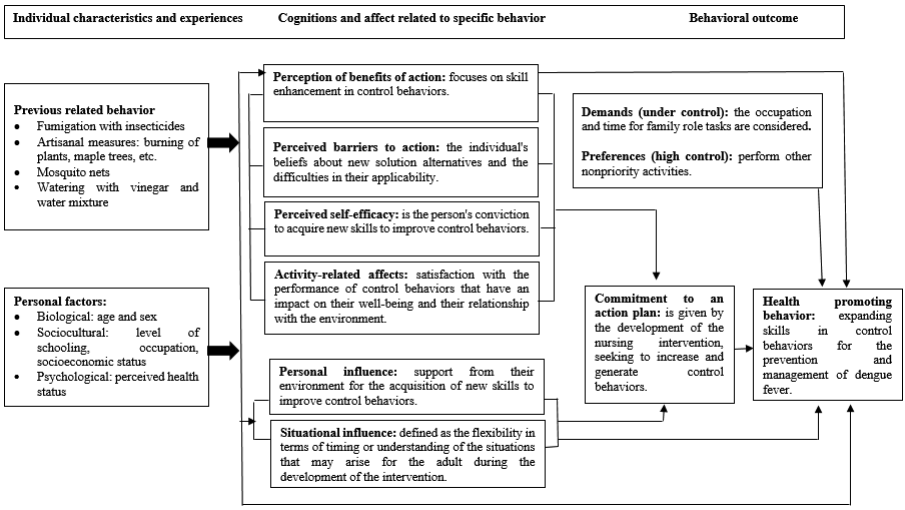
Description of the conceptual elements for the study of health promoting behavior.

From the HPM perspective, the core concepts and subconcepts are used to achieve health-promoting behavior. Once the individual characteristics and experiences, cognitions, and affects related to the specific behavior are identified, the commitment to an action plan is expected, mediated in the research through the development of the nursing intervention that will generate a behavioral outcome, which is considered the expansion of skills in control behaviors in the prevention and management of dengue (Figure 1).

The objective of this study is to evaluate the effect of a nursing educational intervention to promote dengue control behaviors. We hypothesize that the participants in the intervention group will achieve better dengue control behaviors than participants in the usual care group.

## Methods

### Study Design

A randomized controlled trial that follows the guidelines of SPIRIT (Standard Protocol Items: Recommendations for Interventional Trials) is used [23]. The study protocol was registered in ClinicalTrials.gov (NCT05321264).

### Randomization and Blinding

Simple randomization into groups of equal size will be performed using the EPIDAT 4.2 program (Department of Health, Council of Galicia, Spain). Study participants will be randomized 1:1 to receive either the educational intervention or usual care. The randomization and allocation concealment will be carried out by the principal investigator. A centralized telephone randomization system will be used for masking [24]. With respect to blinding, the nurse who will perform the intervention will be blinded and will not know the outcome indicators of the study; the outcome assessors will be blinded and will not know to which group (intervention or control) the participants belong.

### Participants, Setting, and Sample

The study participants are rural residents attending health promotion and disease prevention programs in a health care institution located in the municipality of Montería. It is a city in Colombia and capital of the department of Córdoba. It has 490,935 inhabitants and is considered a cattle-raising town.

Participants will be recruited during the opening hours established by the health institution. A sample size of 116 (58 participants for each group) was calculated according to the following parameters: effect size: an expected difference of 0.5 in the score of the nursing outcome labels between both groups [24], a statistical power of 90%, an α type error of 5%, a SD of the outcome scores of 1.0, an average correlation between the first and second evaluations of 0.3, an intervention/control ratio of 1:1, and a loss adjustment of 20%. Stata (version 16.0; StataCorp) software was used for this purpose.

### Procedures

#### Eligibility Criteria

Persons older than 18 years of age, residing in a rural area of the municipality of Monteria in an endemic area with dengue or with exposure to dengue, attending health promotion and disease prevention programs of a health institution with an initial score ≤a 3.5 in the Nursing Outcome Labels (NOC) risk control (1902) and participation in health care decisions (1606), will be included in the study [25].

#### The Study Intervention

The educational nursing intervention in adults to promote dengue control behaviors, in terms of its structure, will follow the guidelines established by Sidani and Braden [26]. In addition, it will have as a theoretical reference Nola Pender’s HPM, which is articulated for the phenomenon under study, to allow reaching a health-promoting behavior, which lies specifically in the expansion of skills in control behaviors in the face of dengue. The intervention contemplates 3 active ingredients that influence dengue control behavior: an educational component, behavioral support, and attitudinal support (Textbox 1).

**Content of the intervention.**

**Active ingredients and components**

- Session 1: educational component

General information on dengue: definition of the disease, etiological agent, mosquito life cycle, modes of transmission, period of transmissibility, and personal and environmental risk factors (tanks, flower vases, pet drinkers, and plastic containers).

- Session 2: educational component

Dengue overview: health threats, clinical manifestations, diagnostic aids, treatment, prevention and control strategies, and barriers that may prevent the implementation of effective measures.

- Session 3: behavioral support

Commitment to the implementation of preventive strategies (personal, home, and environmental care), achievements obtained with the implementation of dengue strategies, self-control in dengue decision-making, and lifestyle changes.

- Session 4: attitudinal support

Main support networks that people can find in their environment: family, friends, health, and community resources. How to take advantage of available resources?

The intervention will consist of 4 face-to-face meetings with an interval of 1 week for 1 month, each lasting 45 minutes (Textbox 2). The first session will include an educational component and address issues related to dengue, etiological agents, transmission mechanisms, and personal and environmental risk factors. The second session will cover topics such as health threats, clinical manifestations, diagnostic aids, treatment, prevention and control strategies, and barriers that may prevent the application of effective measures against dengue, also belonging to the educational component. The third session will cover personal care practices, home and environment care, and lifestyle modification, which correspond to behavioral support. Final, the fourth session covers attitudinal support, which will review support networks and the use of resources.

**General characteristics of the intervention.**

- Objective: increase knowledge and application of dengue control behaviors.
- Duration of the intervention: 1 month
- Number of sessions: 4 sessions
- Frequency: once a week for a period of 1 month.
- Environment: institution providing health services in rural areas (promotion and prevention program).
- Delivery strategies: individual, face-to-face, standardized educational material, and use of digital tools.
- Receiver: adult who meets inclusion criteria.
- Provider: qualified and trained nurse, with previous performance verification (does not work in health institution).
- Outcome measures: risk control and participation in health care decisions.

The intervention will be delivered in a personalized, face-to-face manner [27], and standardized educational material (booklet) will be used [28-30]. It also indicates that feedback, reviews of key points, and commitments will be made in each session that is held.

#### Intervention Fidelity

The intervention will be delivered to adults as it is designed, seeking to generate the desired changes in the results [26]. For this, it is necessary to develop methodological strategies to ensure the application of the active ingredients (delivery mode, doses, and activities) of the intervention, called fidelity. The fidelity of the intervention contemplates 2 fundamental levels: the theoretical level and the operational level, which are described in Table 1.

**Table 1:** Operationalization of intervention fidelity.

Levels and criteria			Objective		Strategies to ensure fidelity	
**Theoretical**						
	Study design control	Ensure that the intervention is consistent with the theoretical propositions of the Health Promoting Behavior.		The content of the study will be validated by experts to determine consistency between the active ingredients and the proposed theoretical component.		
**Operational**						
	Interventionist training	Ensure that the intervention is delivered in accordance with the protocol.		A course on dengue will be held and nurse training will be provided.		
	Delivery of the intervention	To reduce variability in the application of the intervention.		The intervention will be delivered by a trained nurse and performance will be verified. Development of the intervention protocol manual, with standardized educational material.		
	Treatment exposure	Ensure that all adults receive the intervention on equal terms.		Provide the intervention to all participants as defined in the intervention protocol manual: frequency, dosage, duration, delivery method, and sequence.		
	Treatment reception	Verify the appropriation and use of the active components of the intervention: educational, behavioral, and attitudinal support.		At the end of each session, there will be a space for feedback, review of key points, and commitments.		
	Follow-up of treatment compliance	Verify the implementation of health promoting behavior.		Face-to-face and telephone sessions will be held to provide feedback on the proposed objectives to achieve health-promoting behavior.		

#### Data Collection and Measures

An intervention will be developed in 4 sessions with baseline, end line, and 2-month postintervention measures. The NOC labels chosen to evaluate the effect of the intervention are risk control (1902) and participation in health care decisions (1606).

To characterize the participants in both the intervention and control groups, a form containing variables such as age, sex, education level, marital status, actual occupation, health system affiliation, socioeconomic stratum, and dengue diagnosis will be filled out.

To evaluate the cognitive aspects or mental state of the adult, the mini mental test will be used, which is composed of 30 dichotomous items that evaluate 6 cognitive processes: temporal orientation, spatial orientation, fixation memory, evocation memory, attention and calculation, and language [31,32].

### Outcomes

The outcomes of risk control and participation in health care decisions, which belong to the NOC classification, which is useful for analyzing and measuring the impact and quality of nursing interventions, will be evaluated.

#### Risk Control

Corresponding to code 1902, it consists of 21 indicators on a Likert-type scale with a score from 1 to 5 (1=never demonstrated, 2=rarely demonstrated, 3=sometimes demonstrated, 4=frequently demonstrated, and 5=always demonstrated) and is defined as personal actions to understand, avoid, eliminate, or reduce health threats that are modifiable [33]. It includes items that seek the implementation of behaviors by the adult.

#### Participation in Health Care Decisions

Defined as personal involvement in the selection and evaluation of health care options to achieve a desired outcome, it corresponds to code 1606 and consists of 15 indicators. It uses measurement scale 13, which is defined as the frequency of clarifying by report or behavior and has a score from 1 to 5 (1=never demonstrated, 2=rarely demonstrated, 3=sometimes demonstrated, 4=frequently demonstrated, and 5=always demonstrated) [33]. It contains several indicators that respond to some subconcepts of the theory, such as perceptions of benefits to action, perceptions of barriers to action, and preferences, which are the ones intended to be measured.

### Follow-Up

Participants will be followed up 1 month after the end of the study intervention [34-36]. Individual and face-to-face follow-up is planned to assess the effect of the intervention over time.

### Data Analysis

The information will be tabulated using the EpiData program, and the Stata software will be used for statistical analysis, following the principle of intention-to-treat analysis, which allows the patients to be analyzed as they were originally assigned or randomized [37]. Statistical hypothesis tests will be performed with an α level of 5%. A descriptive analysis of the baseline variables will be made to observe the behavior in each of the groups, using the student 2-tailed *t* test and the Fisher exact test. Categorical variables will be presented with absolute and relative frequencies. Quantitative variables with normal distribution will be presented with mean and SD, and for variables that do not comply with the principle of normal distribution, they will be presented with median and IQR (IQR=Q3–Q1). Normality will be assessed using the Kolmogorov-Smirnov test.

Baseline variables will be compared between the control group and the intervention group. The chi-square test will be used for categorical variables. The student *t* test will be used to compare the means in the 2 groups when the normality assumption is met, and otherwise, the nonparametric Mann-Whitney *U* test will be used.

To control for possible confounding variables, a statistical adjustment will be made through an analysis of covariance with their respective 95% CIs.

The effect of the intervention will be measured through analysis of covariance by means of the delta value or difference of the NOC label score between the 2 comparison groups.

Paired *t* tests will be performed to observe separately how each group fared before and after the intervention and at follow-up.

### Ethical Considerations

The trial will be conducted taking into account the ethical guidelines established in the Declaration of Helsinki, the Belmont Report (1979), the Council for International Organizations of Medical Sciences (2002), and Resolution 8430 of 1993 (Ministry of Health, Colombia, 1993). Ethical approval was granted by the Research Ethics Committee of the Faculty of Nursing of the University of Antioquia (#2021-29). Adults willing to participate voluntarily in the study will be selected, applying the principle of fairness, and the information provided by them will be maintained under strict confidentiality. Alphanumeric codes will be used to record the information, and no names or any other personal identification data will be used. The participants in the control group at the end of the research will receive the same educational intervention as the intervention group.

## Results

It is expected that participants in the nursing education intervention group will have better dengue control behaviors than those in the usual care group (effect size increase of at least 0.5 of the δ score in each of the NOC outcomes). The results are expected to be published in 2025.

## Discussion

### Principal Result

Dengue is linked to household sanitation. The existence of breeding sites is due to specific human behaviors that favor them, whether individual, community, or institutional [15]. Insufficient knowledge about the disease is one of the factors that precipitates the application of inadequate measures [38,39].

Research indicates that risk factors are fostered by the community, largely caused by artificial reservoirs or unprotected tanks in homes [40,41]; it also indicates that the lack of information hinders the identification of symptoms [38] and the poor application of effective measures.

In the study scenario, there is evidence of inadequate knowledge and practices regarding dengue [20], aspects that this study seeks to improve in order to generate favorable changes in the behavior of the population and, therefore, of the disease.

The results of the research may serve as a basis for the identification of lines of action that should be strengthened in the prevention and control of dengue, including the induction and reinduction of the human talent necessary for health care.

Likewise, the research is a window for theoretical-practical integration in health promotion scenarios, and at the same time, it is configured as an opportunity for the visibility of care and its standardization in the rural population. It is hoped that the results will serve to strengthen the role of nursing as an active member of health risk management.

### Limitations

Length of follow-up, cointerventions, and attrition during the study are anticipated limitations. To reduce these limitations, the selection of participants will be controlled by random assignment to the intervention, specific eligibility criteria for entry into the study, and intention-to-treat analysis. Both the intervention and outcome assessors will receive appropriate training. Masking and adjustments to the statistical analysis will be performed as needed. Health care professionals will be sensitized to avoid cointervention in the study. The principal investigator will be the study supervisor and will not be involved in intervention delivery or outcome assessment.

### Conclusion

The scarcity of studies with a high level of evidence, such as randomized clinical trials, provides an opportunity for the development of the present research, which has a quantitative approach and aims to generate new knowledge based on the best scientific evidence for the care of vector-borne diseases in rural populations.

With the evaluation of results through the NOC, the possibility of having measurement indicators for the discipline is opened, which allow making contributions based on evidence, given that the studies carried out show scarce application of the same, so they become important work elements for the development of care processes in favor of the less favored collectivities.

## Appendix

Multimedia Appendix 1 Peer review report from the Universidad de Antioquia, Colombia.

## Abbreviations

HPM: Health Promotion Model
NOC: Nursing Outcome Classification
SPIRIT: Standard Protocol Items: Recommendations for Interventional Trials
