# Supplementary material for: Effectiveness of a Nursing Educational Intervention in Adults to Promote Control Behaviors Against Dengue: Protocol for a Randomized Controlled Trial
Source: JMIR Res Protoc. 2024 Feb 23;13:e54286. doi: 10.2196/54286 (PMC10924258; doi:10.2196/54286)
Supplement: Multimedia Appendix 1 [file resprot_v13i1e54286_app1.pdf]

EL JEFE DEL CENTRO DE INVESTIGACIÓN

HACE CONSTAR:

Que el proyecto de investigación "*Effectiveness of a Nursing Educational Intervention in Adults to Promote Control Behaviors Against Dengue: Study Protocol for a Randomized Controlled Trial"*, fue revisado y aprobado por pares evaluadores externos en el proceso de formación académica del Doctorado en Enfermería de Yolima Judith Llorente Pérez (Investigadora Principal).

Asimismo, la doctoranda Yolima Judith Llorente Pérez recibió financiamiento parcial de $4.471.011 pesos colombianos *(grant # 10410023/ ES84190045-02-2022),* para el desarrollo de la investigación de parte del Grupo de Investigación La Práctica de Enfermería en el Contexto Social, beneficiado de la Convocatoria Estrategia para la Sostenibilidad de Grupos de Investigación de la Universidad de Antioquia 2020-2021, mediante Resolución Rectoral 47573 del 27 de enero de 2021, código ES84190045.

La presente constancia se expide en Medellín-Colombia a los 24 días del mes de agosto de 2023.

Atentamente,

Prof. Wilson Cañon Montañez, PhD
Jefe Centro de Investigación

EVALUADOR 1 - (Reviewer 1)

CRITERIOS DE EVALUACIÓN	Puntaje Máximo	Puntaje Obtenido	OBSERVACIONES
**Planteamiento del problema:** Expone de forma coherente la definición del problema de estudio. Antecedentes, magnitud, preguntas de investigación. Presenta un estado del arte sistemático, riguroso y actualizado donde identifica los vacíos en el conocimiento y la necesidad del estudio.<br>**Impacto y Pertinencia:** Identifica el aporte al conocimiento y a la solución de problemas del conocimiento y de la práctica.	10	7	No son claros los vacíos, presenta una importante pertinencia social pero no es claro en lo disciplinar, dado que el fenómeno de investigación es un tema de interés en salud pública y requiere de una intervención interdisciplinar en la que participa enfermería pero de la que no es exclusiva, así dentro de la relevancia disciplinar no se aborda el rol de enfermería ante esta problemática
**Justificación: Responde de forma directa o indirecta a las siguientes preguntas:**<br>¿La importancia, utilidad e innovación del estudio se encuentra debidamente argumentada?<br>¿Se identifican con claridad los conceptos centrales del estudio?<br>¿Describe el aporte al conocimiento disciplinar y a la solución de problemas de la profesión?	5	3	Desde lo social, es clara la utilidad y pertinencia del proyecto de investigación, no representa mayor innovación y la perspectiva de enfermería desde la promoción de la salud está bien definida, más no el rol dentro del control del dengue, de manera que el solo articular con una teoría no implica que la relevancia disciplinar este presente.

**Referente o marco teórico/conceptual**: **¿L**a teoría y sus conceptos se articulan con el problema que se investiga?, ¿Su formulación es coherente? ¿La perspectiva epistemológica se articula con el problema de investigación y es pertinente? ¿Se presenta el marco teórico o el referente conceptual del estudio articulado con el problema de estudio de manera clara, coherente y sustentada? ¿Presenta una síntesis del campo de la investigación y los abordajes desde diversas disciplinas?	10	7	En la parte final de este documento, se hacen observaciones mayores respecto al marco teórico y la articulación del fenómeno de investigación con la teoría seleccionada.
**Objetivos:** ¿Están vinculados con el problema y las preguntas planteadas? ¿Son viables, claros, concretos y factibles, de acuerdo con el estudio y la metodología?	5	4	Son concretos, coherentes con el problema; sin embargo, requieren revisión respecto a ampliación de los mismos.
**Propuesta metodológica:** ¿Declara explícitamente un enfoque metodológico? ¿Presenta los diferentes elementos de diseño metodológico desde la justificación del diseño, el acceso, las decisiones muéstrales, la recolección de la información, el análisis y el retiro del campo? ¿El diseño metodológico es coherente, viable y factible para lograr los objetivos? ¿El diseño proyectado es riguroso y reconoce las posibles amenazas a la validez? ¿Se justifica epistemológicamente la	10	6	Aunque la metodología está bien definida, se requiere ampliar la justificación respecto al desarrollo de un ECA teniendo en cuenta la realidad en la que se piensa llevar a cabo la intervención. Desde la viabilidad considero que es poco probable que se pueda hacer un control detodas las variables. Se amplía más al final de este documento.

metodología seleccionada?<br>¿Existe coherencia entre enfoque epistemológico y la metodología?			
**Consideraciones éticas:**<br>¿Están contemplados los criterios éticos para garantizar la protección de potenciales participantes?<br>¿Se consideran asuntos de respeto, confidencialidad, beneficencia y no maleficencia?	5	4	Teniendo en cuenta la Resolución 8430 de 1993 declare cuál es el nivel de riesgo de esta investigación y de qué manera piensa manejar este riesgo.<br>Sumando a eso, la población ala que dirige la intervención ¿es potencialmente vulnerable? ¿Cómo considera esto en su proyecto y cómo piensa abordarlo?
**Cronograma y Presupuesto:**<br>¿La secuencia de actividades se adecúa a las fases de desarrollo del proyecto?<br>SI X NO ☐<br><br>¿La duración en cada una de las etapas es apropiada y garantiza el cumplimiento del objetivo?<br>SI ☐ NO X<br><br>¿Los rubros son pertinentes?<br>SI X NO ☐<br><br>¿Los montos son los adecuados para cumplir los objetivos del proyecto?<br>SI ☐ NO X	5	3	Considero que el tiempo para la intervención no es coherente con el grado de complejidad que implica su desarrollo.<br><br>Los valores del presupuesto no son coherentes (Imprevistos, Cartilla y Programa estadístico) y hacen falta aspectos como el costo/hora del asesor y de docentes u otros profesionales que le apoyen en el desarrollo de su proyecto, además de gastos para publicación o apropiación social del conocimiento que están dentro del cronograma pero no en el presupuesto.
**Capacidad de escritura, estilo y normas de escritura científica:**<br>El documento presenta una coherencia adecuada entre sus diferentes apartados,	5	4	En general es un documento bien redactado y cumple con normas de ortografía, no obstante la invitación es a ser concretos en algunos aspectos como el marco teórico y ampliar en otros que lo

estructuración clara de ideas, ortografía y estilo.			requieren como el marco metodológico.
**Relevancia y Pertinencia:** Para evaluar estos aspectos considere los siguientes interrogantes:<br>¿El proyecto permite la generación de conocimiento en la comunidad científica respectiva? O ¿aporta a la resolución de problemas concretos de la realidad?<br>¿La propuesta permite evidenciar algún efecto producido esperado en un grupo, comunidad, institución, etc.?	5	3	El proyecto puede aportar a la solución de un problema real, en especial en poblaciones vulnerables.<br><br>No es claro cómo se pretende evidenciar en la realidad social el efecto de esta intervención, que solo realizará enfermería.
**Originalidad:**<br>Considere si el tema es novedoso, se enmarca en una problemática crucial o importante en la comunidad científica respectiva. Responde a un vacíode conocimiento en el área, si corresponde a un tema que no necesariamente es de frontera a nivel mundial, pero es de gran importancia para el país o si formula nuevas preguntas de investigación.	5	2	No es clara la novedad del mismo por el abordaje de la intervención, dado que desde políticas públicas hay estrategias interdisciplinares implementadas para el control del dengue desde la promoción de la salud.
**Bibliografía referenciada:**<br>¿Son referencias actualizadas y pertinentes para el trabajo realizado?<br>Se presenta bibliografía nacionale internacional además de bibliografía en otros idiomas. Incluye referencias actualizadasy contextualizadas a los últimos 5años. Contiene clásicos referenciados y otros que muestran el estado actual de la cuestión.	5	5	Adecuada referenciación.

**Coherencia interna del trabajo:** La estructura demostrativa o argumentativa se relacionan con la pregunta, el planteamiento del problema, justificación, los objetivos y metodología.	10	8	El proyecto está escrito en forma coherente, sin embargo, la articulación teórica no está bien establecida y el diseño seleccionado, si bien puede funcionar en el papel, no es claro cómo sería viable en la realidad.
**TOTAL**	80	56	Se hacen observaciones adicionales y específicas en la parte final de este documento.

EVALUADOR 2 – (Reviewer 2)

CRITERIOS DE EVALUACIÓN	Puntaje Máximo	Puntaje Obtenido	OBSERVACIONES
**Planteamiento del problema:** Expone de forma coherente la definición del problema de estudio. Antecedentes, magnitud, preguntas de investigación. Presenta un estado del arte sistemático, riguroso y actualizado donde identifica los vacíos en el conocimiento y la necesidad del estudio.<br>**Impacto y Pertinencia:** Identifica el aporte al conocimiento y a la solución de problemas del conocimiento y de la práctica.	10	6	No se evidencia de forma coherente la definición del problema de estudio. Los antecedentes son pobres, poco documentados con evidencia científica y referentes bibliográficos. El estado del arte no es riguroso, ni sistémico. Así como tampoco se muestra el vacío en el conocimiento y la necesidad del estudio. No se reporta el impacto para la práctica de Enfermería
**Justificación: Responde de forma directa o indirecta a las siguientes preguntas:**<br>¿La importancia, utilidad e innovación del estudio se encuentra debidamente argumentada?<br>¿Se identifican con claridad los conceptos centrales del estudio?<br>¿Describe el aporte al conocimiento disciplinar y a la solución de problemas de la profesión?	5	4	En la propuesta se argumenta la utilidad del estudio, se logran visibilizar los conceptos centrales y su aporte al conocimiento disciplinar, se recomienda mejorar la redacción.

**Referente o marco teórico/conceptual**: **¿L**a teoría y sus conceptos se articulan con el problema que se investiga?, ¿Su formulación es coherente? ¿La perspectiva epistemológica se articula con el problema de investigación y es pertinente? ¿Se presenta el marco teórico o el referente conceptual del estudio articulado con el problema de estudio de manera clara, coherente y sustentada? ¿Presenta una síntesis del campo de la investigación y los abordajes desde diversas disciplinas?	10	8	El marco teórico se articula con el problema de investigación, se profundizan los constructos centrales de la investigación. La perspectiva epistemológica se articula con el tema central.
**Objetivos:** ¿Están vinculados con el problema y las preguntas planteadas? ¿Son viables, claros, concretos y factibles, de acuerdo con el estudio y la metodología?	5	5	Los objetivos son claros, factibles, alcanzables y responden a la estructura metodológica.
**Propuesta metodológica**: ¿Declara explícitamente un enfoque metodológico? ¿Presenta los diferentes elementos de diseño metodológico desde la justificación del diseño, el acceso, las decisiones muéstrales, la recolección de la información, el análisis y el retiro del campo? ¿El diseño metodológico es coherente, viable y factible para lograr los objetivos? ¿El diseño proyectado es riguroso y reconoce las posibles amenazas a la validez? ¿Se justifica epistemológicamente la metodológica seleccionada?	10	6	Se declara el enfoque metodológico, debe hacer más claridad en la población de estudio, criterios de selección y de exclusión, y profundizar en el análisis estadístico de la información.

¿Existe coherencia entre enfoque epistemológico y la metodología?			
**Consideraciones éticas:** ¿Están contemplados los criterios éticos para garantizar la protección de potenciales participantes? ¿Se consideran asuntos de respeto, confidencialidad, beneficencia y no maleficencia?	5	5	Están descritos con claridad los criterios éticos, se declaran ampliamente aspectos como: confidencialidad, respeto, beneficencia y no maleficencia. Sobre todo porque es una investigación donde se hará intervención a la población.
**Cronograma y Presupuesto:** ¿La secuencia de actividades se adecúa a las fases de desarrollo del proyecto? SI ☐ NO ☐ ¿La duración en cada una de las etapas es apropiada y garantiza el cumplimiento del objetivo? SI ☐ NO ☐ ¿Los rubros son pertinentes? SI ☐ NO ☐ ¿Los montos son los adecuados para cumplir los objetivos del proyecto? SI ☐ NO ☐	5	4	En el cronograma se detallan cada una de las actividades a considerar en cada etapa de la investigación.<br><br>El presupuesto se cuantifica un poco bajo para el alcance del estudio
**Capacidad de escritura, estilo y normas de escritura científica:** El documento presenta una coherencia adecuada entre sus diferentes apartados, estructuración clara de ideas, ortografía y estilo.	5	3	El documento se podría mejorar en su estructura interna, algunas ideas no son claras. Se recomienda revisar la ortografía y estilo.
**Relevancia y Pertinencia: Para evaluar estos aspectos considere los siguientes interrogantes:** ¿El proyecto permite la generación de conocimiento en la comunidad científica respectiva? o ¿aporta a la resolución de problemas	5	4	El tema propuesto es de interés para la comunidad científica y general. Da respuesta a una gran problemática de la sociedad y sus resultados se podrían generalizar en población similar.<br>El dengue es un problema de salud pública que cada vez

concretos de la realidad?<br>¿La propuesta permite evidenciar algún efecto producido esperado en un grupo, comunidad, institución, etc.?			cobra más vidas, y enfermería desde la promoción y prevención está dada a su intervención
**Originalidad:**<br>Considere si el tema es novedoso, se enmarca en una problemática crucial o importante en la comunidad científica respectiva. Responde a un vacío de conocimiento en el área, si corresponde a un tema que no necesariamente es de frontera a nivel mundial, pero es de gran importancia para el país o si formula nuevas preguntas de investigación.	5	4	El tema es original, se enmarca en una problemática importante para la comunidad científica y general.<br>Desde la formulación del problema se debe hacer visible el vacío del conocimiento que pretende llenar, observándose debilidades en este apartado
**Bibliografía referenciada:**<br>**¿**Son referencias actualizadas y pertinentes para el trabajo realizado?<br>Se presenta bibliografía nacional e internacional además de bibliografía en otros idiomas.<br>Incluye referencias actualizadas y contextualizadas a los últimos 5 años.<br>Contiene clásicos referenciados y otros que muestran el estado actual de la cuestión.	5	4	La bibliografía es pertinente pero debe hacer mayor énfasis en el estado del conocimiento del área de estudio.
**Coherencia interna del trabajo:** La estructura demostrativa o argumentativa se relacionan con la pregunta, el planteamiento del problema, justificación, los objetivos y metodología.	10	7	Existe una coherencia entre los objetivos, pregunta, metodología y referentes conceptuales. Se recomienda mejorar planteamiento y justificación.
**TOTAL**	80	60	

EVALUADOR 3 - (Reviewer 3)

CRITERIOS DE EVALUACIÓN	Puntaje Máximo	Puntaje Obtenido	OBSERVACIONES
**Planteamiento del problema:** Expone de forma coherente la definición del problema de estudio. Antecedentes, magnitud, preguntas de investigación. Presenta un estado del arte sistemático, riguroso y actualizado donde identifica los vacíos en el conocimiento y la necesidad del estudio.<br>**Impacto y Pertinencia:** Identifica el aporte al conocimiento y a la solución de problemas del conocimiento y de la práctica.	10	7	Sugiero mejorar la redacción, signos de puntuación y acentuación. El apartado 1.1.4 debería tratar el papel de enfermería en la promoción de conductas de control del dengue en la población adulta rural.
**Justificación: Responde de forma directa o indirecta a las siguientes preguntas:**<br>¿La importancia, utilidad e innovación del estudio se encuentra debidamente argumentada?<br>¿Se identifican con claridad los conceptos centrales del estudio?<br>¿Describe el aporte al conocimiento disciplinar y a la solución de problemas de la profesión?	5	3	Sugiero mejorar la redacción de la justificación. De forma más concreta presentar la importancia, utilidad e innovación del estudio. Discurre sobre aspectos que no son relevantes para mostrar la importancia del estudio; así como, evidenciar el aporte al conocimiento disciplinar y a la solución de problemas de la profesión.

**Referente o marco teórico/conceptual**: **¿L**a teoría y sus conceptos se articulan con el problema que se investiga?, ¿Su formulación es coherente? ¿La perspectiva epistemológica se articula con el problema de investigación y es pertinente? ¿Se presenta el marco teórico o el referente conceptual del estudio articulado con el problema de estudio de manera clara, coherente y sustentada? ¿Presenta una síntesis del campo de la investigación y los abordajes desde diversas disciplinas?	10	8	Falta mayor claridad en la articulación del problema de estudio y la teoría de Nola Pender. Sugiero desarrollar la estructura teórico conceptual empírica solo para el ECC. Luego, en otro apartado, describir la teorización que se hace en el estudio para el explicar el efecto de la intervención.
**Objetivos:** ¿Están vinculados con el problema y las preguntas planteadas? ¿Son viables, claros, concretos y factibles, de acuerdo con el estudio y la metodología?	5	4	Deben vincularse con la población rural. Los específicos están redactados en forma de actividades.
**Propuesta metodológica**: ¿Declara explícitamente un enfoque metodológico? ¿Presenta los diferentes elementos de diseño metodológico desde la justificación del diseño, el acceso, las decisiones muéstrales, la recolección de la información, el análisis y el retiro del campo? ¿El diseño metodológico es coherente, viable y factible para lograr los objetivos? ¿El diseño proyectado es riguroso y reconoce las posibles amenazas a la validez? ¿Se justifica epistemológicamente la metodológica seleccionada?	10	7	Sí cumple con todos los aspectos; no obstante, no es clara la descripción de la primera fase del estudio dado que presenta la validación del resultado, pero no de la intervención. Tampoco queda claro si la intervención ya fue elaborada. En caso de que no se haya elaborado aún la intervención, es necesario describir os pasos que se seguirán para su elaboración. En el apartado del ensayo clínico mencionar que estrategias se utilizarán para prevenir las pérdidas de los participantes y cómo se ocultará la randomización.

¿Existe coherencia entre enfoque epistemológico y la metodología?			
**Consideraciones éticas:**<br>¿Están contemplados los criterios éticos para garantizar la protección de potenciales participantes?<br>¿Se consideran asuntos de respeto, confidencialidad, beneficencia y no maleficencia?	5	5	
**Cronograma y Presupuesto:**<br>¿La secuencia de actividades se adecúa a las fases de desarrollo del proyecto?<br>SI NO x<br>¿La duración en cada una de las etapas es apropiada y garantiza el cumplimiento del objetivo?<br>SI x NO ¿Los rubros son pertinentes?<br>SI x NO ¿Los montos son los adecuados para cumplir los objetivos del proyecto?<br>SI NO x	5	3	Al leer el documento, no es claro si ya se elaboró o no la intervención. En contraste, al leer el cronograma, pareciera que la intervención ya se elaboró. Debe adicionar otros rubros al presupuesto como lo son papelería, consulta bases de datos, etc. El costo de una investigación como la propuesta por el estudiante supera el monto mencionado en el presupuesto.
**Capacidad de escritura, estilo y normas de escritura científica:** El documento presenta una coherencia adecuada entre sus diferentes apartados, estructuración clara de ideas,<br>ortografía y estilo.	5	2	Sugiero revisar la redacción de todo el documento debido a que falta precisión en muchas de las ideas formuladas, las líneas que componen los párrafos son muy extensas (aspecto que le resta claridad a las ideas), hay faltas de puntuación y acentuación.
**Relevancia y Pertinencia: Para evaluar estos aspectos considere los siguientes interrogantes:**<br>¿El proyecto permite la generación de conocimiento en la comunidad científica respectiva? o ¿aporta a la resolución de problemas	5	5	

concretos de la realidad?<br>¿La propuesta permite evidenciar algún efecto producido esperado en un grupo, comunidad, institución, etc.?			
**Originalidad:**<br>Considere si el tema es novedoso, se enmarca en una problemática crucial o importante en la comunidad científica respectiva. Responde a un vacío de conocimiento en el área, si corresponde a un tema que no necesariamente es de frontera a nivel mundial, pero es de gran importancia para el país o si formula nuevas preguntas de investigación.	5	5	
**Bibliografía referenciada:**<br>**¿**Son referencias actualizadas y pertinentes para el trabajo realizado?<br>Se presenta bibliografía nacional e internacional además de bibliografía en otros idiomas. Incluye referencias actualizadas y contextualizadas a los últimos 5 años.<br>Contiene clásicos referenciados y otros que muestran el estado actual de la cuestión.	5	5	
**Coherencia interna del trabajo:** La estructura demostrativa o argumentativa se relacionan con la pregunta, el planteamiento del problema, justificación, los objetivos y metodología.	10	7	
**TOTAL**	80	61	
